# Revealing Measles Outbreak Risk With a Nested Immunoglobulin G Serosurvey in Madagascar

**DOI:** 10.1093/aje/kwy114

**Published:** 2018-06-06

**Authors:** Amy K Winter, Amy P Wesolowski, Keitly J Mensah, Miora Bruna Ramamonjiharisoa, Andrianmasina Herivelo Randriamanantena, Richter Razafindratsimandresy, Simon Cauchemez, Justin Lessler, Matt J Ferrari, C Jess E Metcalf, Jean-Michel Héraud

**Affiliations:** 1 Department of Epidemiology, Johns Hopkins Bloomberg School of Public Health, Baltimore, Maryland; 2 Princeton Environmental Institute, Princeton University, Princeton, New Jersey; 3 Virology Unit, Institut Pasteur de Madagascar, Antananarivo, Madagascar; 4 Mathematical Modeling of Infectious Diseases Unit, Institut Pasteur, Paris, France; 5 Intercollege Graduate Degree Program in Ecology, Pennsylvania State University, University Park, Pennsylvania; 6 Department of Ecology and Evolutionary Biology, Princeton University, Princeton, New Jersey

**Keywords:** Madagascar, measles, rubella, serological survey, surveillance

## Abstract

Madagascar reports few measles cases annually and high vaccination campaign coverage. However, the underlying age profile of immunity and risk of a measles outbreak is unknown. We conducted a nested serological survey, testing 1,005 serum samples (collected between November 2013 and December 2015 via Madagascar’s febrile rash surveillance system) for measles immunoglobulin G antibody titers. We directly estimated the age profile of immunity and compared these estimates with indirect estimates based on a birth cohort model of vaccination coverage and natural infection. Combining these estimates of the age profile of immunity in the population with an age-structured model of transmission, we further predicted the risk of a measles outbreak and the impact of mitigation strategies designed around supplementary immunization activities. The direct and indirect estimates of age-specific seroprevalence show that current measles susceptibility is over 10%, and modeling suggests that Madagascar may be at risk of a major measles epidemic.

Measles is a highly infectious disease that is preventable with a safe, effective, and inexpensive vaccine (1). The World Health Organization (WHO) African Region aims to eliminate measles by 2020 (2, 3). Many of these countries currently achieve high vaccination coverage rates via routine and supplementary immunization activities (SIAs)—periodic vaccination campaigns for targeted age ranges (2). Nonetheless, measles transmission continues, and the WHO African Region has the highest reported incidence, with 27.9 incident cases per 1 million population in 2016 (2).

Routine measles immunization began in Madagascar in 1985. Although vaccination coverage remained low through the 1980s and 1990s (4), progress in reducing measles incidence has occurred since 2004, in part as a result of regularly implemented SIAs occurring at 3–5 year intervals (5). Measles cases reported to the WHO show a sharp drop since 2004, from tens of thousands of cases annually to fewer than 10 cases per year (6). Madagascar is one of 19 WHO African Region countries where minimal surveillance targets have been met, including investigating ≥2 cases of non-measles febrile-rash illness per 100,000 population annually and obtaining a blood specimen from ≥1 suspected measles case in ≥80% of districts annually (2).

Measles incidence in Madagascar is reportedly low, SIA coverage is reportedly high, and surveillance targets are being met; however, uncertainty as to the success of the measles program remains. Settings with low transmission and low routine coverage (<95%), such as Madagascar, may experience a “honeymoon period” in which individuals who were missed by vaccination programs escape infection as a result of reduced transmission, and thus susceptible individuals can accumulate (7). If additional vaccination campaigns are not routinely implemented, this accumulation of susceptible individuals may exceed a critical threshold, which can result in disease outbreaks (7, 8). Identifying a country’s risk for a measles outbreak following a period of low transmission requires knowledge of the underlying age profile of susceptibility, which shapes outbreak potential.

Readily available epidemiologic data, including vaccination coverage and case surveillance data, can be used to indirectly infer population immunity. However, inaccurate or incomplete data sources can result in biased estimates of susceptibility (9–11). Outbreaks have occurred after the “honeymoon period” in several WHO African Region countries despite low estimates of susceptibility based on high reported vaccination coverage (12–15). To mitigate issues associated with indirect estimates of population susceptibility, serological surveys (the detection of antigen-specific antibodies in a population) can provide a direct measurement of immunity (16, 17) and may refine estimates of measles age-specific seroprevalence.

The age profile of immunity can be used to characterize a country’s risk of a measles outbreak, but achieving this also requires accounting for potentially complex transmission dynamics and their variability across age. Mathematical models are a powerful tool for exploring outbreak risk and optimal design for vaccination mitigation strategies (18–20), by allowing us to track individual movement through age or epidemiologic stages while accounting for nonlinearities in transmission dynamics that might be counterintuitive (21).

In the present study, we conducted a nested immunoglobulin G (IgG) serological survey by testing existing serum samples from the febrile-rash surveillance system in Madagascar to directly measure the age distribution of immunity (16, 17). Given the potential for external validity bias, because the serum samples were passively collected, we assessed sampling variability according to age and space, and we compared our direct estimates of the age-specific profile of immunity from serological data with indirect estimates based on a birth cohort projection method that takes into account natural and vaccine-derived immunity (22). Building on these direct estimates of the age profile of immunity, we used an age-structured mathematical model to explore the capability of different SIA strategies, including the most recent SIA administered, to contain the risk of a measles outbreak in Madagascar.

## METHODS

### Data collection and testing protocol

Serum samples were obtained from Madagascar’s national surveillance system for measles and rubella. Standard protocol requires that when a patient presents for care with symptoms meeting the clinical criteria for measles (fever and rash and either cough, corzya, or conjunctivitis), the patients’ serum is collected and sent to the WHO national reference laboratory located at the Institut Pasteur de Madagascar. At Institut Pasteur de Madagascar, the serum is tested for measles-specific and rubella-specific immunoglobulin M antibodies to detect a recent infection. Any remaining serum is stored at Institut Pasteur de Madagascar.

In 2016, we tested serum collected between November 2004 and November 2015 for measles-specific IgG antibodies with an indirect ELISA test (Enzygnost Anti-Measles Virus/IgG; Siemens, Erlangen, Germany). IgG antibodies are a marker of past exposure (either to measles natural infection or vaccination) and represent immunity. Quantitative results, in mIU/mL, were obtained from IgG testing, based on a sensitivity of 90% and specificity of 100% (23). Measles seropositivity was defined as IgG antibody concentration greater than 200 mIU/mL per assay manufacturer’s instructions. Due to potential nonrandom misclassification bias related to serum testing error and waning humoral immunity (i.e., cell-mediated immunity may still play a role even if an individual tests seronegative (24)), seroprevalence does not perfectly map to measles immunity. However, to simplify the analysis we assumed that these were equal.

### Data sample size

We excluded the febrile-rash serum samples collected prior to and during the October 2013 SIA (November 2004–October 2013) in order to estimate the most recent age-specific seroprofile and avoid biasing our estimates of age-specific seroprevalence due to sharp shifts in seroprevalence among the 2013 SIA-targeted age groups. A total of 1,123 samples were collected from November 2013 to December 2015. By grouping the recent 26 months, we assume monotonic increases in immunity (natural and vaccinal) over age during this time; this assumption likely holds for natural immunity given low transmission (2) as well as for vaccinal immunity, because vaccination coverage is unlikely to have declined substantially in this time-frame, so our analysis should be robust to this assumption.

Of the 1,123 samples collected between November 2013 and December 2015, 20 samples did not contain enough serum to test for measles IgG antibodies, and were removed from the analysis. Samples were also removed if the patients’ age in years was unknown (3 samples). We removed 17 samples that tested measles immunoglobulin M positive to reduce oversampling of measles seropositive individuals via natural infection that resulted from sampling via febrile-rash surveillance. Samples considered IgG-antibody equivocal (antibody concentration between 100 and 200 mIU/mL) were retested; those that remained equivocal (78 samples) were removed from the analysis, although we also conducted a sensitivity analysis assuming equivocal tests were seronegative. A total of 1,005 samples were used to estimate current age-specific seroprevalence.

### Characterization of the data

Identifying sampling biases by age and location is key to understanding the external validity of the data to characterize age-specific seroprevalence. We compared the proportion of samples according to age with the expected proportion according to United Nations Population Division estimates (25). We also investigated the degree to which the 1,005 febrile-rash samples were generalizable across space, and we assessed the potential of heterogeneous spatial sampling to bias our estimates of measles seroprevalence by comparing spatial variation in sampling with spatial variation in inferred vaccinal immunity. We did not assess potential spatial variation in naturally acquired immunity because: 1) Evidence suggests that recent transmission is low; 2) we removed immunoglobulin M measles-positive cases to reduce this bias; and 3) the immunoglobulin M incidence data is insufficient in sample size to assess spatial differences.

### Estimates of proportion seropositive according to age

We directly estimated seroprevalence according to age from the IgG serological data using a nonparametric model with local polynomial estimators, given its flexibility in allowing nonmonotonicity (“locfit” library (26) in R (R Foundation for Statistical Computing, Vienna, Austria)) (16) (see Web Appendix 1, Web Figure 1, available at https://academic.oup.com/aje for details). The estimated total population proportion seropositive was age-adjusted using Madagascar’s 2015 population age structure (25).

We compared our direct empirical estimates of age-specific seroprevalence with indirect estimates of the age profile of immunity. The indirect method, developed by Takahashi et al. (22), estimates the proportion of each birth cohort that is immune based on routine immunization, SIAs, and natural infection. We estimated the proportion of each birth cohort vaccinated from each vaccination opportunity, routine vaccination or SIA, according to WHO coverage estimates (4), and estimated the fraction of natural infection over time from estimated measles incidence extracted from Simons et al. (27) (see Web Appendix 2, Web Tables 1 and 2, Web Figure 2 for details).

### Exploring the effect of measles SIA scenarios

We assessed the impact of 16 different SIA scenarios by simulating the administration of each SIA (taking into account vaccination effectiveness) to Madagascar’s population, assuming age-specific susceptibility reflected the direct estimates based on the serological data. SIA scenarios differed according to the targeted upper age and the assumed vaccination coverage. We included the age group that was targeted by the 2016 SIA, which was conducted after data collection (ages 9 months through 4 years) (5); a typically targeted age group (ages 9 months through 14 years); and 2 nonclassically targeted age groups (ages 9 months through 9 years and 9 months through 19 years). For each scenario, we analyzed an upper and lower bound of potential vaccination campaign coverage (70% and 95%, based on Madagascar’s reported range from WHO (5), but setting the maximum to 95% despite reports exceeding 100% due to known overreporting issues using administrative data (10)). Simulated SIAs assumed independence between SIA vaccination and prior immunity (i.e., all individuals in the target age range had an equal probability of being vaccination by the SIA regardless of immunity status). We assumed that SIA vaccine effectiveness by age followed a logistic function modeled on data from Boulianne et al. (28), saturating at 97%, and that there was no correlation between prior immunity and SIA vaccination.

We used 4 approaches to evaluate the impact of each SIA scenario. First, we compared estimated population susceptibility levels after the SIA with the theoretical susceptibility threshold for elimination. In an unstructured population, the critical immunity threshold $\left(p_{c}\right)$ required to achieve herd immunity is defined as $p_{c}=1-(1/R_{0})$ (29), where *R*_0_ is the basic reproductive number evaluated at 10, 15, and 20 (30); the susceptibility threshold is its complement $\left(1-p_{c}\right)$. The critical immunity threshold does not account for the pattern of transmission and immunity over age. Therefore, we additionally estimated the effective reproductive number, *R*_eff_, after the SIA (assuming assortative age contacts following Mossong et al. (31)) and compared this with the elimination threshold of 1 (30). *R*_eff_ is the average number of secondary cases per typical infected individual, here estimated using next-generation techniques to account for age-specific patterns (32).

The third SIA evaluation approach compared the estimated number of measles cases after the SIA with the estimated number of cases for the condition in which no SIA was implemented. We used a discrete-time, deterministic, age-structured mathematical model to simulate Madagascar’s measles transmission dynamics, with and without SIA, following the introduction of an infected individual. Finally, we estimated the number of vaccine doses needed to vaccinate 1 susceptible individual, accounting for vaccine effectiveness according to Boulianne et al. (28) and accounting for the fact that SIAs target all individuals within an age target regardless of their immunity status. See Web Appendices 3 and 4 for details on scenario evaluation and the age-structured model, respectively.

## RESULTS

### Data characterization

**Figure 1.**
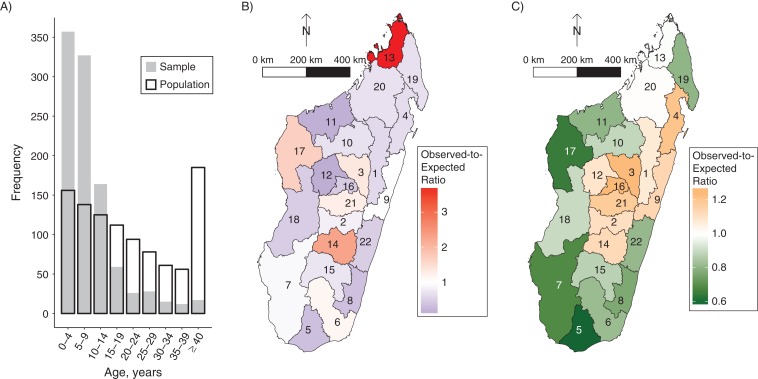
Febrile-rash surveillance serological samples according to age and region, Madagascar, 2013–2015. A) The population and sample age structure in 5-year age groups. Sample age distribution is represented by the grey bars (*n* = 1,005), and the population age distribution (based on UNPD data (25)) is represented by the black-border bars. Younger persons (ages <15 years) are overrepresented in the sample. B) The sampling ratio of observed samples to expected samples (i.e., the proportion of samples from each region (*n* = 1,005) divided by the proportion of Madagascar’s population that resides in each region (44, 45)). The regions in red tints were oversampled. C) The vaccination ratio of observed to expected measles vaccination coverage (i.e., measles vaccination coverage by region divided by the national measles vaccination coverage, which is 0.69 (4, 46)). The central plateau and some eastern regions reported the highest vaccination coverage.

Of the 1,005 samples, we found that individuals less than 15 years old were oversampled (Figure 1A), likely because age is significantly associated with measles seroprevalence (33). Estimated total population immunity was accordingly adjusted for age.

Figure 1B shows evidence of spatial sampling bias: Some regions (tinted red) were oversampled and other regions (tinted purple) undersampled. However, we found that variation in vaccination coverage was unlikely to be an important driver of variation in the number of samples per region because the sampling ratio by region (Figure 1B) and the vaccination ratio by region (Figure 1C) were not correlated (see Web Appendix 5, Web Figure 3).

### Age-specific measles susceptibility profiles

**Figure 2.**
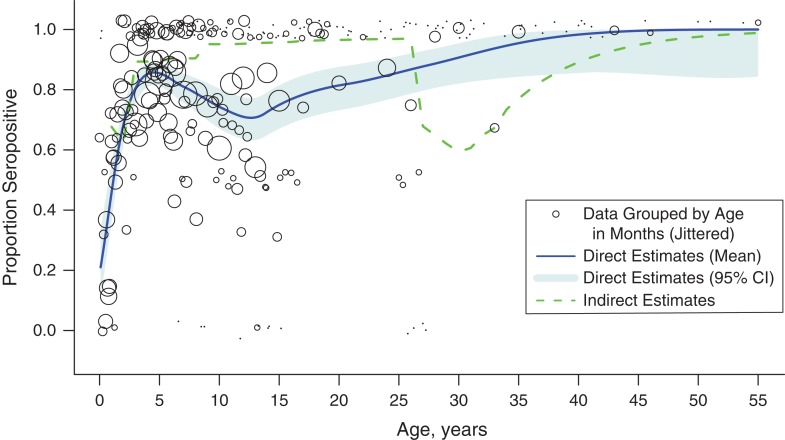
Direct and indirect estimates of proportion measles seropositive by age, Madagascar, 2013–2015. The black dots represent the observed seroprevalence according to age in months based on the nested serological data (*n* = 1,005), where the size of the dots corresponds to the number of samples for each age in months. The blue solid and light blue shaded areas represent the mean and 95% confidence interval (CI) of the directly estimated proportion seropositive, based on the serological data, and show a general increase in seroprevalence with age, with the exception of a dip around age 13 years. The dashed green line represents the indirect estimates of proportion seropositive according to age, based on the birth cohort projection method. The 4 supplementary immunization activities (SIAs) conducted prior to 2015, including a 2004 SIA that targeted children ages 9 months through 14 years, had a large impact on indirectly inferred immunity (see Web Appendix 1 for history of SIAs). The indirect method estimated high immunity in children and adults up to age 25 years and a large pocket of susceptibility for those ages just outside the 2004 SIA targeted age group.

Direct and indirect estimates of immunity differed over age (Figure 2; see Web Appendix 6 and Web Figure 4 for sensitivity analysis assuming equivocal tests were seronegative). The blue line and shaded light blue area represent the mean and 95% confidence interval of direct estimates of the age profile of immunity fitted to serological data. Over age 40 years, everyone sampled tested seropositive for measles. Prior to this age, there was a general increase in the proportion of individuals seropositive over age with an exception around age 13 years, where there was a drop in immunity. The indirect method, represented by the green dashed line, estimated 95% immunity among ages 8–26 years, as a result of routine measles vaccination and frequent SIAs, and a large pocket of susceptible individuals aged 27–32 years. This encompasses individuals who were not eligible for any SIAs, who were exposed to low estimated routine coverage during childhood and, for those individuals missed by the routine vaccine, were potentially also exposed to low measles transmission throughout their childhood and adolescence via indirect protection (see Web Appendix 2 for details on how past transmission was discounted during vaccination years).

**Figure 3.**
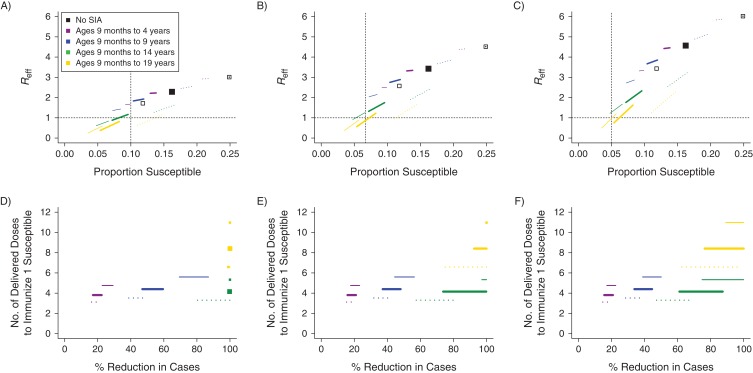
The impact of supplementary immunization activity (SIA) on proportion susceptible, effective reproductive number (*R*_eff_), campaign efficiency, and outbreak size, using data from Madagascar, 2013–2015. Using an age-structured model informed by the direct age-specific seroprevalence estimates, we simulated the impact of each SIA. We compared the proportion of the population susceptible (*x*-axis) to *R*_eff_ (*y*-axis) for assumed *R*_0_ values of 10 (A), 15 (B), and 20 (C), 1 week following the implementation of measles SIA scenarios (age targets: lower age of 9 months through upper ages of 4, 9, 14, and 19 years). For reference, the black dashed lines indicate the thresholds of *R*_eff_ = 1 and susceptibility threshold (1 − *p*_*c*_). We found that for all values of *R*_0_, broader age ranges (i.e., up to ages 14 or 19 years) were necessary to achieve *R*_eff_ less than 1. If *R*_0_ was 10 or 15, we found disagreement between the 2 evaluated elimination thresholds (proportion susceptible <(1 − *p*_*c*_) and *R*_eff_ < 1) in their evaluation of the impact of SIA scenarios. We compared the estimated percent reduction in the number of measles cases over time (*x*-axis) and campaign efficiency (number of doses delivered to successfully vaccinate 1 susceptible individual; *y*-axis) for assumed *R*_0_ values of 10 (D), 15 (E), and 20 (F), across measles SIA scenarios (age targets: lower age of 9 months through upper ages of 4, 9, 14, and 19 years). We found that a SIA campaign that targeted children of ages through 14 years to be the most efficient at deploying dosages to susceptible individuals while also reducing a large percentage of cases. Colors indicate the campaign age targets, including the condition in which no SIA was conducted. Thick lines indicate the expectation for the directly estimated mean age profile, and solid and dotted thin lines correspond to the upper and lower confidence bounds, respectively, of the directly estimated age profile. Lines span the range of outcomes for coverage rates of 70%–95% (right to left on the lines) for each SIA.

Despite differences in direct and indirect estimates of immunity by age, both suggested overall high population susceptibility in Madagascar as of 2015. Using the direct method, we estimated that 83.2% of Madagascar’s current population was immune to measles (95% confidence interval: 74.7, 87.7). The indirect estimate of the proportion immune (88.9%) fell just outside the 95% confidence interval. Both imply that population immunity in Madagascar is below the simple unstructured estimate of the critical threshold for herd immunity (90%–95%) (3). Additionally, direct estimates of *R*_eff_ surpass the elimination threshold of 1 (range, 1.7–6.0; Figure 3A–3C, black squares). These results suggest that Madagascar may be at risk of a significant measles outbreak (34, 35).

### The effect of measles SIA scenarios

The SIA conducted in late 2016 among children aged up to 4 years may have been insufficient to reduce the risk of a measles outbreak in Madagascar given that our estimates of *R*_eff_ remains above 1 after the SIA (Figure 3A–3C, purple lines). The model indicates that, if a measles outbreak were to start, measles cases would be reduced by at most 29.2% with the designed SIA, compared with the condition in which no SIA was administered. Overall, extending the vaccination campaign to include more ages would result in a larger reduction in the proportion of susceptible individuals and a lower *R*_eff_ (Figure 3A–3C). Our analysis suggests that SIA campaigns targeting children aged 9 months old through 14 years best balance cases prevented per vaccine dose administered and total case numbers; this would successfully vaccinate a susceptible person per every 3–5 doses delivered and would reduce the number of measles cases in a measles outbreak by between 66% and 100%, depending on *R*_0_ and the true starting age immunity profile (Figure 3D–3F). This finding is the result of the unusual gap in measles immunity directly estimated between ages 10 and 15 years (Figure 2). These results were qualitatively robust to assuming an age-contact matrix reflecting the 6 countries assessed in the European POLYMOD diary study (31).

Assuming that *R*_0_ was 20 resulted in general agreement between the 2 elimination thresholds (proportion susceptible $<\left(1-p_{c}\right)$ and *R*_eff_ < 1) in their evaluation of the impact of SIA scenarios (Figure 3C). However, for lower values of *R*_0_ (10 and 15), we found scenarios in which the estimated *R*_eff_ was greater than 1 after the SIA, even though the population proportion susceptible was less than $\left(1-p_{c}\right)$ after the SIA (Figure 3A and 3B).

## DISCUSSION

Over the past decade, Madagascar has reported low numbers of measles cases and high SIA vaccination coverage, but how this translates into population immunity and its pattern over age has not, to our knowledge, previously been described. Serological data can be used to directly estimate the age-specific seroprevalence and discern whether susceptibility remains below the theoretical critical threshold to sustain elimination or whether the population is at risk of a measles outbreak (16, 36, 37). In the present study, we leveraged existing serum samples by conducting a nested serosurvey within Madagascar’s febrile-rash surveillance system, avoiding the financially and logistically challenging features of a nationally representative serosurvey (38). Age-structured mathematical models, in combination with age-specific seroprevalence estimates, were then used to explore the impact of different SIA designs to contain the risk of a measles outbreak (39, 40).

Neither our direct or indirect estimates can be validated as true; the former is affected by the fact that a sample based on passive febrile-rash surveillance is potentially nonrepresentative, and the latter is affected by uncertainty and known biases in vaccination coverage data (10) and incidence data (27). However, both the indirect and direct estimates suggested that Madagascar may be at risk of a measles outbreak (29, 34, 35, 41).

The differences in the age profiles of immunity between the methods highlight gaps in our understanding of vaccination programs. In particular, direct estimates obtained from the serological data suggested that the 2004 SIA was heterogeneously applied across ages, an important and yet rarely described feature of this type of intervention. Peak vaccination coverage of the 2004 SIA appears to have been achieved for children whose age was around the middle of the target age range, and was lower for children at the bottom and top of the target age ranges. The direct estimates also showed no increased probability of being immune in children targeted by the 2007 SIA, potentially suggesting overestimated SIA coverage. Madagascar experienced a political crisis in 2009 that may have hindered routine measles vaccination, potentially explaining the beginning of a decline in immunity after age 5 years as of 2015.

Our analysis also provides an important window into evaluating recent measles immunization policy. Madagascar conducted a measles SIA in October 2016 among children aged 9 months through 59 months, with reported 96% coverage (5). While our model assumed spatial homogeneity in *R*_0_ and that individuals were well-mixed across Madagascar (given that necessary data to estimate this was not available), so that measles outbreak size will be overestimated, differences relative to the absence of a campaign are likely to be broadly informative of SIA impact. Our results suggested that the 2016 SIA was insufficient to reduce the risk of a measles outbreak given high estimated susceptibility among ages outside the target age range. Rather, SIAs targeting children through age 14 years would further decrease potential outbreak size while successfully vaccinating the most susceptible individuals per dose delivered. Fortunately, there is an opportunity to conduct a campaign of this magnitude in the near future. Madagascar is considering introduction of the rubella-containing vaccine which, if eligible for financial support from Gavi, the Vaccine Alliance, would include a measles-rubella vaccination campaign targeting children aged 9 months through 14 years (42).

In order to explore the relevance of heterogeneous age immunity and transmission on the effect of SIAs, we compared 2 different elimination thresholds (proportion susceptible $<\left(1-p_{c}\right)$ and *R*_eff_ < 1) and found that age-specific transmission increasingly affected outbreak potential as measles transmission (*R*_0_) decreased. In Madagascar, evidence suggests that the *R*_0_ for measles may in fact be low (10–15), given high estimated measles susceptibility and inferred low transmission. Therefore, age-specific transmission may play a significant role in understanding and maintaining measles elimination in Madagascar. This result may also be more broadly applicable in settings where successful vaccination programs have reduced measles transmission, such that finer-scale age-contact data and analysis may be required to achieve appropriate targeting as countries move closer to elimination.

There are a number of limitations and assumptions in this analysis. The nonprobability sample via the febrile-rash passive surveillance system may misrepresent measles seroprevalence at the country level. For example, following vaccination, up to 5% of individuals may experience transient fever or rash (43), such that febrile-rash surveillance may oversample measles-vaccinated individuals, biasing seroprevalence estimates upwards. Nongeneralizability of serological samples collected and sent for testing may also play a role in biasing estimates of measles immunity. A problematic systematic bias might emerge if health-care workers used knowledge of a patients’ past vaccination record (via recall or vaccination card) to inform their diagnosis and decision to collect and send serological samples, rather than abiding by the febrile-rash surveillance guidelines. This would result in fewer samples from vaccinated individuals and a potential overestimation of measles susceptibility in Madagascar.

In this analysis, we corrected for age sampling bias by age-adjusting estimates of population susceptibility. However, we are unable to correct for age bias if undersampling of older individuals resulted in insufficient power to correctly estimate measles immunity for these ages. We additionally assessed the potential for spatial sampling bias on estimates of the age profile of vaccinal immunity. We found that the febrile-rash surveillance system sampled vaccination coverage and connected areas representatively across regions on average, suggesting this may not be an important source of bias. To our knowledge, the present study is the first to demonstrate the use of a nested IgG serosurvey within febrile-rash surveillance systems to assess population immunity. However, much work remains to assess the external validity of this data source.

Measles IgG serological surveys provide a direct measure of an age immunity profile to identify susceptible ages and inform the need for SIAs. Despite age-specific differences in seroprevalence, both our direct and indirect estimates of total proportion immune suggested high measles susceptibility as of 2015. Building on these direct estimates by modeling measles transmission dynamics and their variability across age, our simulated results suggest that Madagascar is at risk of a measles outbreak. Madagascar’s measles control program must remain vigilant to reduce the number of susceptible individuals via vaccination and reduce the risk of outbreaks.

## Supplementary Material

Web Material

## Abbreviations

IgG: Immunoglobulin G
SIA: supplementary immunization activities
WHO: World Health Organization
