# Supplementary material for: Revealing Measles Outbreak Risk With a Nested Immunoglobulin G Serosurvey in Madagascar
Source: Am J Epidemiol. 2018 Jun 6;187(10):2219–26. doi: 10.1093/aje/kwy114 (PMC6166215; doi:10.1093/aje/kwy114)
Supplement: Web Material [file kwy114_winter_web_material_final.pdf]

Web Material for "Revealing Measles Outbreak Risk with a Nested IgG Serosurvey in Madagascar"

Web Appendix 1. Direct Estimates of Proportion Seropositive by Age

We directly estimated the proportion of seropositive individuals by age from the IgG serological data using a non-parametric model with local polynomial estimators, given its flexibility in allowing non-monotonic functions over age. We used a nearest neighbor generalized cross validation method in selecting the optimal value for the smoothing parameter [1]. Web Figure 1 shows the GCV minimum at 0.65; therefore we set the local polynomial alpha option to 0.65, which means that when fitting the seroprevalence curve at each age, the local fit incorporates 65% of the data, or the closest neighbors. We additionally specified the degree of the local polynomial to 2, i.e., a local quadratic fit. The following line of code was specified using the 'locfit' library in R [2], *locfit(Measles.IgG.binary ∼ Age.in.Months, alpha=0.65, degree=2, data=serodata, family="binomial")*.

Web Appendix 2. Indirect Estimates of Proportion Seropositive by Age

We compared our direct empirical estimates of the age profile of immunity to indirect empirical estimates of the age profile of immunity, based on previously developed methods by [3]. The indirect method estimates the proportion immune for each birth cohort based on its experience of routine immunizations, SIAs, and natural infection. For example, if 80% of the birth cohort was routinely vaccinated and the cumulative measles attack rate was 75% among those unvaccinated, then 95% of the cohort would be estimated as

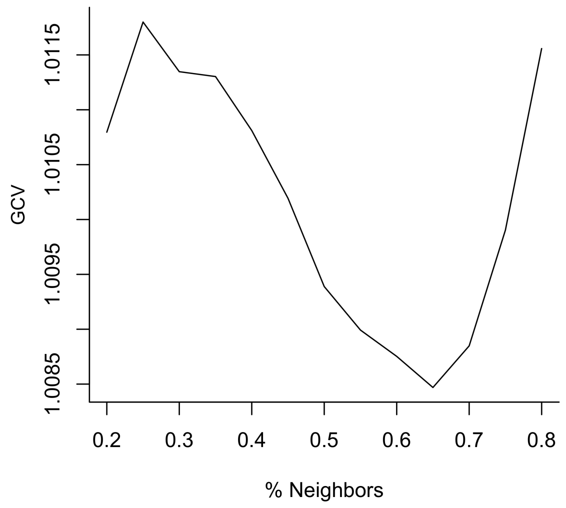

Web Figure 1: GCV curve for the nearest neighbor approach

immune [3]. While this example showcases the method, it is also a simplification, assuming 100% vaccine effectiveness by age, a time constant cumulative attack rate, and no opportunity for SIAs; all of these issues are encompassed in the method described in further detail below.

We assumed that routine vaccination coverage rates were equivalent to the World Health Organization (WHO) and United National Children's Fund (UNICEF) estimates for routine MCV1 coverage rates for Madagascar between years 1985 to 2014 [4]. SIA timing, age ranges, and vaccination coverage rates were extracted from WHO reported administrative estimates [5]. Madagascar administered four measles SIAs prior to 2015 (Web Table 1). Because all SIAs took place in the month of October, the SIA coverage rate contributed to theoretical estimates of the proportion immune for the year following the SIA. We assumed complete overlap between the probability of routine and SIA vaccination, so that where a cohort had experienced vacci-

nation via an SIA as well as routine vaccination, coverage in that cohort was taken as be the highest of the two values. We assumed a maximum coverage rate of 95% in both SIAs and routine immunization, and applied a vaccination effectivness rate over age based on [6] to SIA and routine vaccination coverage rates.

Web Table 1: Measles SIAs conducted in Madagascar 2004-2015, based on WHO reported administrative estimates [5]

Date	Targeted Age Range	Reported Admin Coverage	Reported Survey Coverage
October 2004	9 mo - 14 yo	99%	NA
October 2007	9 mo - 4 yo	100%	67%
October 2010	9 mo - 3 yo	93%	NA
October 2013	9mo - 4yo	92%	84%

The probability of natural immunity as a function of age was estimated by assuming a constant hazard of infection for ages 1 to 100 years old. This is only an approximation, for younger ages the effect of natural immunity will be outweighed by the effect from vaccination. The base hazard rate for measles endemic years (prior to 1985) was set at 0.149, such that 95% of infections occur prior to 20 years of age [3]:

$$P(\text{infection by age a}) = 1 - exp(-0.149 \times a) \tag{1}$$

The base hazard rate in subsequent years, after the measles vaccine was introduced, was then scaled relative to the proportional decline in estimated measles incidence in each year relative to the mean incidence between 1981 and 1984, e.g., hazard rate in year t = 0.149 × (incidence in year t / mean incidence in 1981 to 1984). Estimated measles incidence was extracted from the 2013 WHO measles burden estimates per [7]. The probability of natural immunity for the first year of life was modified to account for maternal immunity, such that the mean of $(exp(-0.45 * a))$ individuals were considered immune, where a is age in months from 1 to 12 [8].

Web figure 2 display the results of the indirect birth cohort analysis broken down by vaccination and naturally acquired immunity and susceptibility by age.

Both indirect and direct estimation techniques described above provide an estimate of the profile of immunity by age, however, indirect estimates require extrapolation from often poorly resolved parameters such as vaccination coverage; and further, unlike direct estimates, provide no clear measure of uncertainty. A summary of the differences, strengths, and limitations between these two estimation techniques can be found in Web Table 2.

Web Table 2: Comparison between indirect and direct empirical estimates of proportion seropositive by age

	Indirect Estimates	Direct Estimates
Data source(s)	vaccination coverage overtime (routine and SIAs)<br>maternal immunity<br>vaccine effectiveness<br>attack rate overtime	measles IgG serological survey nested within measles/rubella surveillance system
Estimation Technique	combine birth cohort projection of immunity estimates based on each cohort's experience of routine immunizations, SIAs, and natural infection	semiparametric approach using penalized regression smoothers
Limitations	relies of assumed parameters [9]	non-probability sample lacking external validity
Strengths	based on public access data	probabilistic estimates with standard errors,<br>low cost extension to current surveillance system as compared to population-based serological survey
References	[3]	

Web Appendix 3. Exploring the Effect of Measles SIA Scenarios

To evaluate the impact of each SIA scenario we i) estimated population susceptibility levels post SIA and compared it to the susceptibility threshold, $(1 - p_c)$, ii) estimated the effective reproductive number, R_{eff}, post SIA and compared it to elimination threshold of 1, iii) estimated cases post SIA and compared it to the estimated cases if no SIA was implemented, and iv) the estimated the number of vaccine doses needed to vaccinate one susceptible

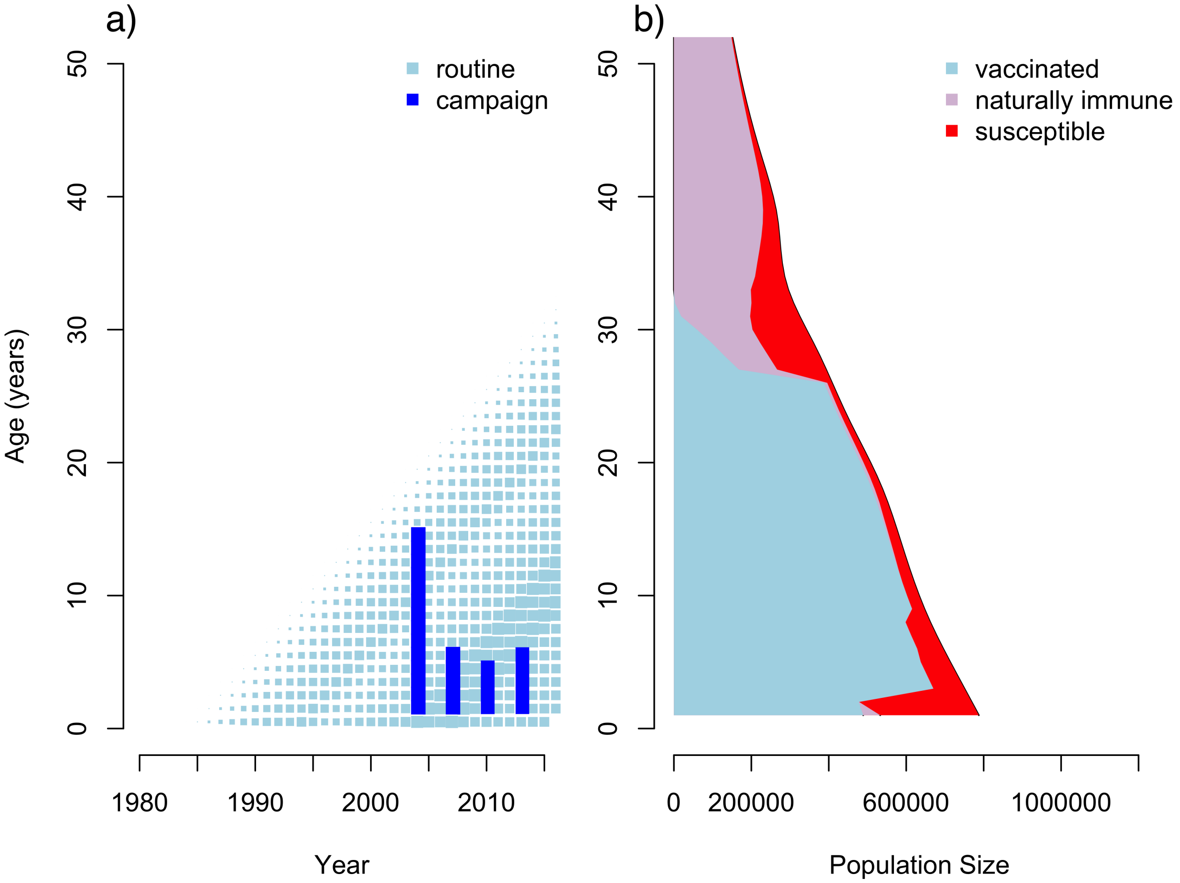

Web Figure 2: Indirect estimates of proportion measles seropositive by age using the birth cohort analysis per [3]. a) Estimated shadow of protection from routine and SIA vaccination across age cohorts. b) Estimated 2015 Malagasy population susceptible, vaccinated, and immune via natural infection.

individual.

The population proportion susceptible was estimated as an age-weighted average of the proportion susceptible immediately following a SIA. The critical immunity threshold (p_c) is defined as $p_c = 1 - (\frac{1}{R_0})$ [10], such that the susceptibility threshold is its complement $(1 - p_c)$. R_0 is the basic reproductive number and is qualitatively defined as the average number of people a typical infected individual will infect in a fully susceptible population [11]. Estimates of R_0 for measles range globally from 5 to 20 [11]; we conducted a sensitivity analysis for three different R_0 values (10, 15, and 20), such that the susceptibility threshold $(1 - p_c)$ was estimated as 10%, 6.7%, and 5%. In the simplest analysis, p_c, is the level of population immunity required to achieve herd immunity [10]; if population immunity is maintained below the critical immunity threshold over time, then the virus has the potential for elimination [12, 10, 13]. While p_c, as defined here, is used as a benchmark for informing policies aimed at elimination [14], in reality, the exact threshold for elimination will be sensitive to the details of the pattern of transmission over age, age structure, and spatial heterogeneity in transmission [15, 12].

The effective reproductive number, R_{eff}, is a related measure used for assessing elimination, but takes into account transmission over age. R_{eff} is the average number of secondary cases per typical infected individual [11]. We estimated R_{eff} using the proportion susceptible immediately following a SIA. We relied on next generation methods to estimate R_{eff} as the dominant eigenvalue of the product of the who acquires infection from whom matrix (pattern of transmission over age) and the proportion susceptible in each age group [16]. We assumed an age-structured mixing process consistent with patterns observed in the POLYMOD diary study across Europe [17]. These age-contact rates were then scaled by a constant value to achieve R_0 equal to the assumed level of transmission we were analyzing (10, 15, and 20). The

R_{eff} must remain below 1 in order to eliminate measles [11]. Given that R_{eff} estimates take into account the pattern of transmission over age unaccounted for in the proportion of the population immune, we directly compared the two elimination thresholds (proportion susceptible $< (1 - p_c)$ and $R_{eff} < 1$).

Lastly, we estimated measles outbreak size post SIA by simulating measles transmission dynamics following the introduction of infected individual using a discrete-time deterministic age-structured mathematical model (previously described in [18]), and compared it to the outbreak size if no SIA was implemented. To estimate outbreak size, we simply aggregated the number of simulated measles cases that occurred within three years of the introduction of an infected individual. We additionally used the model to estimate the number of dosages delivered to successfully vaccinate one susceptible individual, assuming a vaccination effectiveness rate over age per [6], and saturating at 97%. See Web Appendix 4 below for model details.

Web Appendix 4. Simulating Measles Dynamics

Building on our direct estimates of the current landscape of immunity over age, we deterministically simulated measles dynamics in Madagascar for 10 years to assess the impact of SIAs on measles outbreak size. We simulated nine different Malagasy populations based on three different starting populations (mean and upper and lower bound of the 95% CI based on the penalized regression smoothing model as described in main text), and three different levels of measles transmission (R_0 values of 10, 15, and 20). We administered the SIA at time point 1, and we introduced an infected individual of age 10 years old (the age with the highest assumed contact rates) at time point 2 in order to investigate the magnitude of a measles outbreak.

Web Appendix 4.1. Model Structure

To simulate measles transmission dynamics, we used a discrete-time model that incorporates both epidemic and demographic transitions, building on theory introduced in [19, 16]. The model structure described here is as originally presented in [20, 21]. We structured the population into 5 epidemiological stages (maternally immune 'M', susceptible 'S', infected 'I', recovered 'R', and vaccinated 'V', taken to indicate the effectively vaccinated), and 265 age classes (monthly strata up to age 15 years to capture monthly immunity and vaccination rates during early years of life, and yearly strata until age 100 years). The key feature of the model is a large matrix that at every time-step defines transitions from every possible epidemiological stage and age class combination, to every other possible epidemiological stage and age class combination.

We take a two step approach to describe the large transition matrix here. Step one is to define epidemiological transitions, ignoring demographic transitions (aging and survival). Matrix $\mathbf{A}_{a,t}$, captures transitions between each epidemiological stage within each age class a and time-step t, where the time-step t was set to the approximate generation time of measles at about 2 weeks (i.e., there are 24 infection generations per year). Matrix $\mathbf{A}_{a,t}$ is defined as,

$$\mathbf{A}_{a,t} = \begin{bmatrix} 1-d_a & 0 & 0 & 0 & 0 \\ d_a & 1-\varphi_a(\mathbf{n}(t))(1-v_a) & 0 & 0 & 0 \\ 0 & \varphi_a(\mathbf{n}(t))(1-v_a) & 0 & 0 & 0 \\ 0 & 0 & 1 & 1 & 0 \\ 0 & v_a & 0 & 0 & 1 \end{bmatrix}$$

The five rows from left to right and columns from top to bottom represent the 'M', 'S', 'I', 'R', and 'V' epidemiological stages, respectively. In the transition matrix, d_a is the probability of losing maternal immunity by age class a, φ_a

is the probability an individual in age class a becomes infected, and v_a is the probability an individual in age class a is successfully vaccinated. The probability of infection, Φ (also called the force of infection, FOI) is a function of $\mathbf{n}(t)$, a vector describing the population at time t, defined as,

$$\mathbf{n}(t) = (M_{1,t}, S_{1,t}, I_{1,t}, R_{1,t}, V_{1,t}, M_{2,t}, ...V_{z,t})^T \tag{2}$$

according to

$$\varphi_a(\mathbf{n}(t)) = 1 - \exp\left(\frac{-\sum_j \beta_{a,j,t} I^{\gamma}_{j,t}}{\sum \mathbf{n}(t)}\right) \tag{3}$$

where z is the total number of age classes (here z=265), $\beta_{a,j}$ is the rate of transmission between individuals in age class a and j, as known as the Who-Aquires-Infection-From-Whom (WAIFW) matrix, and $I^{\gamma}_{j,t}$ is the number of infected individuals in age class j and time-step t, while γ captures the non-modeled heterogeneities in age mixing [22, 23] and the effects of discretization of the underlying continuous process [24]. In this analysis, we fixed γ at 0.97 [22], because discrete-time models that do not incorporate this exponent result in unrealistically unstable dynamics prone to frequent extinction. Given that measles transmission is frequency dependent, we divided the number of infected individuals in each age class by the total population size [25].

The second step is to construct the full transition matrix, $\mathbf{A}(\mathbf{n}(t))$, that includes both epidemiological transitions captured in matrix $\mathbf{A}_{a,t}$, and demographic transitions (aging and survival). This matrix is used to project the entire population forwards via aging, mortality, and infection dynamics and is defined as,

$$\mathbf{A}(\mathbf{n}(t)) = \begin{bmatrix} s_1(1-u_1)\mathbf{A}_{1,t} & 0 & 0 & ... & 0 \\ s_1u_1\mathbf{A}_{1,t} & s_2(1-u_2)\mathbf{A}_{2,t} & 0 & ... & 0 \\ 0 & s_2u_2\mathbf{A}_{2,t} & s_3(1-u_3)\mathbf{A}_{3,t} & ... & 0 \\ ... & ... & ... & ... & 0 \\ 0 & 0 & 0 & ... & .s_z\mathbf{A}_{z,t} \end{bmatrix}$$

where s_a is the probability that an individual in age class a survives to the next time step, u_a is the rate of aging out of age class a, and $\mathbf{A}_{1,t}$,$\mathbf{A}_{2,t}$, etc., are the defined in equation 4.1. We assumed constant rate of aging into the next age class (i.e., u_a = 1 / (length of age class a in years * 24)). The dynamics of the whole population can then be projected forward in time, such that

$$\mathbf{n}(t+1) = \mathbf{A}(\mathbf{n}(t))\mathbf{n}(t) + \mathbf{B}_t \tag{4}$$

where $\mathbf{B}_t$ is a vector representing the number of births at time t, defined as,

$$\mathbf{B}_t = (b_t, 0, 0, ...0)^T. \tag{5}$$

Web Appendix 4.2. Epidemiological Model Parameters

The duration of protection by measles maternal antibodies ranges between 3 and 9 months [26, 27]; accordingly, we modeled the probability of remaining in the maternally immune epidemiological stage over age $(1 - d_a)$ as an exponential decay function with a constant rate of 0.95 per month.

R_0 is the basic reproductive number and is qualitatively defined as the average number of people a typical infected individual will infect in a fully susceptible population. Estimates of R_0 for measles range globally from 5 to 20 [11]; we assumed three different R_0 values (10, 15, and 20). Transmission

to individuals in age class a, from individuals in age class j are defined by $\beta_{a,j}$. Empirical estimates from large-scale qualitative studies generally converge on assortative age-contact patterns relevant for infections transmitted via the respiratory route, specifically among school ages [17, 28, 29]. Accordingly, we assumed age-specific contact patterns proportional to those characterized in the European POLYMOD study based on diary entries [17] (i.e., high rates of contact among individuals of the same ages and between parent and child ages, a pattern that has also been reported in China and Vietnam [28, 30]). We calculated the WAIFW matrix (i.e., $\beta_{a,j}$) by rescaling the Great Britain age contact rates observed in the POLYMOD study to reflect the assumed R_0 of measles (10, 15, and 20). We additionally conducted a sensitivity analysis of age-contacts using the remaining five country's age contact matrices per the POLYMOD study [17] .

The probability of effective vaccination for individuals in age class a (i.e., v_a) was obtained by multiplying the probability of access to vaccination by age (probability of access to routine vaccination is based on estimates from Zambia [8]; probability of access to SIA vaccination was assumed to be 1 for all ages in the target age range), by a vaccination effectiveness rate over age (based on [6]). The probability of access to routine vaccination over age was scaled to reflect our assumed coverage. And, we forced vaccine effectiveness over age based on [6] to saturate at 97%. The predicted level of routine vaccination coverage for measles containing virus dose one was 64% in Madagascar in 2014 [4], and was held constant over the 10 year simulation time period.

Web Appendix 4.3. Demographic Model Parameters

We extracted the 2015 total population size and population age structure from the United Nations' estimates for Madagascar [31]. We then applied each estimated age immunity profile (mean and upper and lower bound of

the 95% CI based on the penalized regression smoothing model as described above) to the population age structure in order to determine the number of susceptible and immune (i.e. recovered) individuals per age strata as our starting population. Additional data inputs required by the age-structured models included Madagascar's birth and survival rates. Population survival rates per age class were estimated by matching the region's 2013 estimated life expectancy at birth (65 years old; [32]), to the United Nations 'Coale-Demeny East' life table model [33]. We assumed constant rates of mortality by age over the 10 year simulation time period. Crude birth rates between 2015 and 2025 were extracted from the United Nations Population Divisions probabilistic population projection mean estimates for Madagascar [34]. The number of births per time-step t (i.e., b_t) were estimated by multiplying the estimated birth rate per time-step t by the total population at time-step t ($\mathbf{n}(t)$).

Web Appendix 5. Spatially Characterizing the Data

We assessed the febrile-rash surveillance serological samples by region. Figure 1B and 1C in the main text display the sampling ratio of observed to expected samples and the vaccination ratio of observed to expected measles vaccination coverage, respectively. We assessed if sampling was associated with vaccination coverage at the region level by estimating the association between sampling ratios and vaccination ratios; we did not find a significant correlation (Pearson's correlation coefficient: 0.148, p = 0.511), see Web Figure 3.

Web Appendix 6. Sensitivity Analysis of Equivocal Results

In the main text we do not assume to know the serostatus of individuals whose serum tested equivocal. However, we ran a sensitivity analysis where

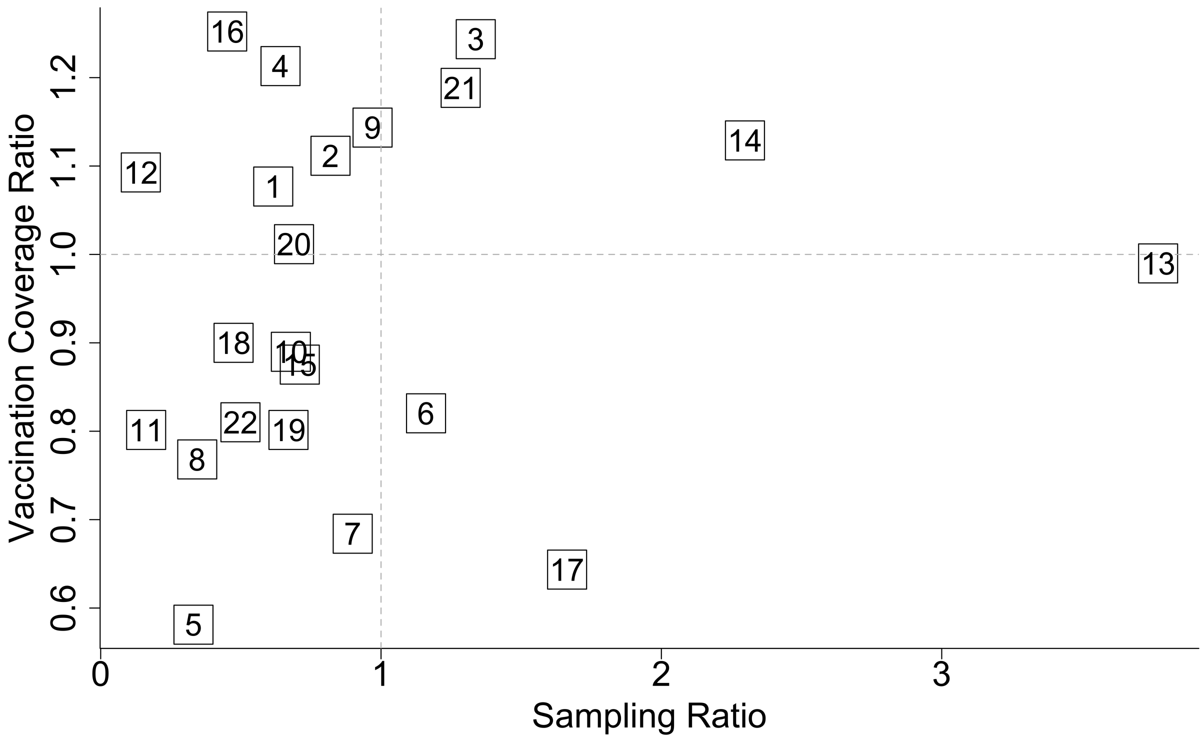

Web Figure 3: Sampling ratio per the nested IgG serosurvey in the febrile-rash surveillance by vaccination coverage ratio by region in Madagascar.

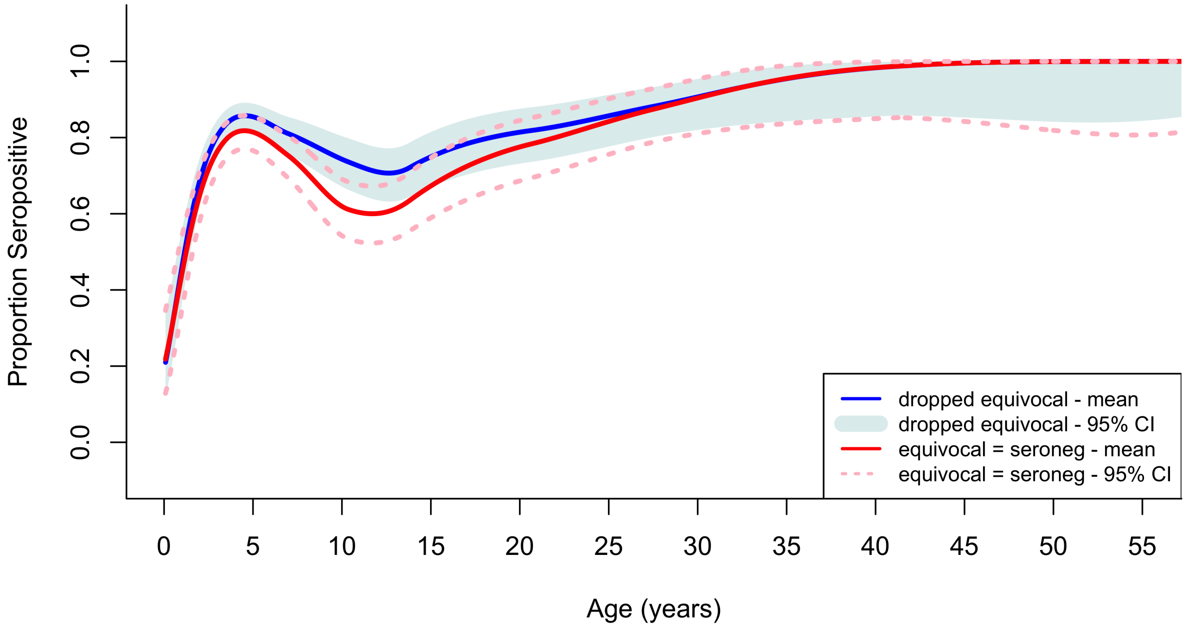

Web Figure 4: Direct estimates of proportion measles seropositive by age. The blue solid and light blue shaded areas represents the mean and 95% confidence interval of the directly estimated proportion seropositive based on the serological data where we dropped all equivocal test results from the analysis (N=1005). The red solid line and pink dashed lines represent the mean and 95% confidence interval of the directly estimated proportion seropositive based on the serological data where we assumed all equivocal test results were seronegative (N=1083).

we coded all 78 equivocal samples as seronegative, or unprotected from infection. Web Figure 4 shows, as expected, that recoded equivocal test results to be seronegative resulted in a lower proportion of seropositive individuals, particularly among children ages 5-20 years old. The effect of an SIA on this age profile of immunity exaggerated the positive benefits of an SIA that targeted children up to 15 years old, results not shown.

References

[1] N. Hens, Z. Shkedy, M. Aerts, C. Faes, P. Van Damme, and P. Beutels. *Modeling infectious disease parameters based on serological and social contact data : a modern statistical perspective*. Statistics for Biology and Health. Springer, New York, 2012.

[2] C. Loader. *Local Regression and Likelihood*. Springer, New York, 1999.

[3] S. Takahashi, C.J. Metcalf, M.J. Ferrari, W.J. Moss, S.A. Truelove, A.J. Tatem, B.T. Grenfell, and J. Lessler. Reduced vaccination and the risk of measles and other childhood infections post-ebola. *Science*, 347(6227): 1240–1242, 2015.

[4] World Health Organization and UNICEF. Estimates of national immunization coverage (WUENIC), estimates for 1980 to 2015, 2017.

[5] World Health Organization. Retrospective measles data on supplementary immunization activities, 2000-2016, 2017.

[6] N. Boulianne, G. De Serres, S. Ratnam, B.J. Ward, J.R. Joly, and B. Duval. Measles, mumps, and rubella antibodies in children 5-6 years after immunization: effect of vaccine type and age at vaccination. *Vaccine*, 13 (16):1611–6, 1995.

[7] E. Simons, M. Ferrari, J. Fricks, K. Wannemuehler, A. Anand, A. Burton, and P. Strebel. Assessment of the 2010 global measles mortality reduction goal: results from a model of surveillance data. *Lancet*, 379 (9832):2173–2178, 2012.

[8] J. Lessler, W.J. Moss, S.A. Lowther, and D.A. Cummings. Maintaining high rates of measles immunization in Africa. *Epidemiology and Infection*, 139(7):1039–1049, 2011.

[9] J. Lessler, C.J.E. Metcalf, R.F. Grais, F.J. Luquero, D.A.T. Cummings,

and B.T. Grenfell. Measuring the performance of vaccination programs using cross-sectional surveys: A likelihood framework and retrospective analysis. *PLoS Medicine*, 8(10), 2011.

[10] P.E.. Fine. Herd immunity: history, theory, practice. *Epidemiologic Reviews*, 15(2):265–302, 1993.

[11] R.M. Anderson and R.M. May. *Infectious Diseases of Humans: Dynamics and Control*. Oxford University Press, New York, 1991.

[12] R.M. Anderson and R.M. May. Age-related changes in the rate of disease transmissions - implications for the design of vaccination programs. *Journal of Hygiene*, 94(3):365–436, 1985.

[13] W.O. Kermack and A.G. McKendrick. Contribution to the mathematical theory of epidemics. *Proceedings of the Royal Society of London. Series A - Containing Papers of a Mathematical and Physical Character*, 115 (772):700–721, 1927.

[14] N. J. Gay. The theory of measles elimination: Implications for the design of elimination strategies. *Journal of Infectious Diseases*, 189:S27–S35, 2004.

[15] N.J. Gay, L.M. Hesketh, P. Morgancapner, and E. Miller. Interpretation of serological surveillance data for measles using mathematical-models - implications for vaccine strategy. *Epidemiology and Infection*, 115(1): 139–156, 1995.

[16] P. Klepac and H. Caswell. The stage-structured epidemic: linking disease and demography with a multi-state matrix approach model. *Theoretical Ecology*, 4(3):301–319, 2011.

[17] J. Mossong, N. Hens, M. Jit, P. Beutels, K. Auranen, R. Mikolajczyk, M. Massari, S. Salmaso, G. S. Tomba, J. Wallinga, J. Heijne, M. Sadkowska-Todys, M. Rosinska, and W. J. Edmunds. Social contacts

and mixing patterns relevant to the spread of infectious diseases. *PLoS Medicine*, 5(3):381–391, 2008.

[18] C.J.E. Metcalf, J. Lessler, P. Klepac, F. Cutts, and B.T. Grenfell. Impact of birth rate, seasonality and transmission rate on minimum levels of coverage needed for rubella vaccination. *Epidemiology and Infection*, 140(12):2290–2301, 2012.

[19] P. Klepac, L.W. Pomeroy, O.N. Bjornstad, T. Kuiken, A.D.M.E. Osterhaus, and J.M. Rijks. Stage-structured transmission of phocine distemper virus in the dutch 2002 outbreak. *Proceedings of the Royal Society of London. Series B (Biological Sciences)*, 276(1666):2469–2476, 2009.

[20] C.J.E. Metcalf, J. Lessler, P. Klepac, A. Morice, B.T. Grenfell, and O.N. Bjornstad. Structured models of infectious disease: Inference with discrete data. *Theoretical Population Biology*, 82(4):275–282, 2012.

[21] J. Lessler and C.J.E. Metcalf. Balancing evidence and uncertainty when considering rubella vaccine introduction. *PLoS One*, 8(7):e67639, 2013.

[22] O.N. Bjornstad, B.F. Finkenstadt, and B.T. Grenfell. Dynamics of measles epidemics: Estimating scaling of transmission rates using a time series sir model. *Ecological Monographs*, 72(2):169–184, 2002.

[23] B.F. Finkenstadt and B.T. Grenfell. Time series modelling of childhood diseases: a dynamical systems approach. *Journal of the Royal Statistical Society Series C-Applied Statistics*, 49:187–205, 2000.

[24] K. Glass, Y. Xia, and B.T. Grenfell. Interpreting time-series analyses for continuous-time biological models-measles as a case study. *Journal of Theoretical Biology*, 223(1):19–25, 2003.

[25] C.J.E. Metcalf, C.V. Munayco, G. Chowell, B.T. Grenfell, and O.N. Bjornstad. Rubella metapopulation dynamics and importance of spatial coupling to the risk of congenital rubella syndrome in Peru. *Journal of the Royal Society Interface*, 8(56):369–376, 2011.

[26] S. Waaijenborg, S.J.M. Hahn, L. Mollema, G.P. Smits, G.A.M. Berbers, F.R.M. van der Klis, H.E. de Melker, and J. Wallinga. Waning of maternal antibodies against measles, mumps, rubella, and varicella in communities with contrasting vaccination coverage. *Journal of Infectious Diseases*, 208(1):10–16, 2013.

[27] C. Nicoara, K. Zach, D. Trachsel, D. Germann, and L. Matter. Decay of passively acquired maternal antibodies against measles, mumps, and rubella viruses. *Clinical and Diagnostic Laboratory Immunology*, 6(6): 868–871, 1999.

[28] P. Horby, Q.T. Pham, N. Hens, T.T. Nguyen, Q.M. Le, D.T. Dang, M.L. Nguyen, T.H. Nguyen, N. Alexander, W.J. Edmunds, N.D. Tran, A. Fox, and T.H. Nguyen. Social contact patterns in Vietnam and implications for the control of infectious diseases. *PLoS One*, 6(2):e16965, 2011.

[29] F. DeStefano, M. Haber, D. Currivan, T. Farris, B. Burrus, B. Stone-Wiggins, A. McCalla, H. Guled, H. Shih, P. Edelson, and S. Wetterhall. Factors associated with social contacts in four communities during the 2007-2008 influenza season. *Epidemiology and Infection*, 139(8):1181–90, 2011.

[30] J.M. Read, J. Lessler, S. Riley, S.Y. Wang, L.J. Tan, K.O. Kwok, Y. Guan, C.Q. Jiang, and D.A.T. Cummings. Social mixing patterns in rural and urban areas of southern China. *Proceedings of the Royal Society of London. Series B (Biological Sciences)*, 281(1785), 2014.

[31] United Nations. World population prospects: The 2015 revision, DVD edition, 2015.

[32] The World Bank Group. Data: Life expectancy at birth, total (years), 2015. URL `http://data.worldbank.org/indicator/SP.DYN.LE00.IN`. Accessed: August 30, 2016.

[33] UN Population Division. World population prospects 2012: Ex-

tended model life tables, 2010. URL http://esa.un.org/wpp/Model-Life-Tables/data/MLT_UN2010-130_1y.xls. Accessed: June 5, 2014.

[34] United Nations and Department of Economic and Social Affairs and Population Division. World population prospects. Probabilistic projections, 2015.
